# How Structure Defines Affinity in Protein-Protein Interactions

**DOI:** 10.1371/journal.pone.0110085

**Published:** 2014-10-16

**Authors:** Ariel Erijman, Eran Rosenthal, Julia M. Shifman

**Affiliations:** Department of Biological Chemistry, The Alexander Silberman Institute of Life Sciences, The Hebrew University of Jerusalem, Jerusalem, Israel; University of Queensland, Australia

## Abstract

Protein-protein interactions (PPI) in nature are conveyed by a multitude of binding modes involving various surfaces, secondary structure elements and intermolecular interactions. This diversity results in PPI binding affinities that span more than nine orders of magnitude. Several early studies attempted to correlate PPI binding affinities to various structure-derived features with limited success. The growing number of high-resolution structures, the appearance of more precise methods for measuring binding affinities and the development of new computational algorithms enable more thorough investigations in this direction. Here, we use a large dataset of PPI structures with the documented binding affinities to calculate a number of structure-based features that could potentially define binding energetics. We explore how well each calculated biophysical feature alone correlates with binding affinity and determine the features that could be used to distinguish between high-, medium- and low- affinity PPIs. Furthermore, we test how various combinations of features could be applied to predict binding affinity and observe a slow improvement in correlation as more features are incorporated into the equation. In addition, we observe a considerable improvement in predictions if we exclude from our analysis low-resolution and NMR structures, revealing the importance of capturing exact intermolecular interactions in our calculations. Our analysis should facilitate prediction of new interactions on the genome scale, better characterization of signaling networks and design of novel binding partners for various target proteins.

## Introduction

Many biological processes are governed by non-covalent interactions between two or more proteins. Binding affinities of functional protein-protein interactions (PPIs) span more than nine orders of magnitude, from very weak and transient interactions observed frequently in signal transduction and membrane trafficking [1], [2] to very strong interactions exhibited by several enzyme-inhibitor complexes with binding affinities (K_d_) reaching 10^−14^ M [3], [4]. In spite of numerous studies that analyze various PPIs [5], [6], precise features that distinguish weak PPIs from high-affinity PPIs remain elusive [7]. An accurate understanding of these interactions would allow us to predict new PPIs on the genome scale, to better characterize known signaling networks, and to study PPI evolution. In addition, it would enable rational design of novel binding interactions, facilitating the discovery of therapeutic molecules that target disease-associated PPIs.

Binding interfaces of protein-protein complexes serve a dual role in protein function, since they can exist both as surfaces of monomeric proteins and as the area buried upon complex formation. They hence should differ in chemical properties from both protein surfaces and protein cores [8]. With the appearance of high-resolution structures for the first protein-protein complexes, a number of groups calculated and analyzed various structure-based features of binding interfaces such as solvent accessible surface area, packing, hydrogen bond (H bonds) and salt bridge patterns and arrived at a number of conclusions (see [9] for a review). Janin and colleagues reported that an interface covering about 1500 Å^2^ and containing at least ten hydrogen bonds possesses sufficient enthalpy to generate binding affinities of up to 10^−14^ M [10]. Xu et al, established that hydrogen bonding geometry at interfaces is less optimal and exhibits wider distribution compared to that at the interior of globular proteins [11]. Thornton and colleagues concluded that interfaces of permanent complexes are more closely packed and contain fewer intermolecular hydrogen bonds compared to interfaces of non-obligatory complexes [5].

The growing number of high resolution structures for various PPIs as well as experiments documenting binding strengths stimulated several investigations directed at predicting PPI binding affinities from structure-derived features [12]–[18]. These works utilized either empirical scoring functions or knowledge–based potentials that both highly depend on the quality of the dataset. The described studies were performed using small datasets where good correlation with binding affinity was observed. However, when evaluated on larger datasets the same methods frequently fail [19]. Recent community-wide assessment of methods for predicting protein binding affinities demonstrated that even distinguishing between native PPIs and computationally designed PPIs that do not bind each other in reality is a difficult task [20].

Prediction of PPI binding affinity faces several limitations. First, binding affinity data is not always reliable and is frequently not compatible between various experiments due to different conditions and experimental techniques used for the measurement. For example, a K_d_ for the same protein-protein complex reported by different groups could easily range by two orders of magnitude (e.g. from 10^−10^ to 10^−12^ for the fasciculin/acetylcholinesterase complex [21]–[23]). An additional inaccuracy in affinity prediction might come from crystal structures that are solved at low or medium resolution and thus might misrepresent certain intermolecular interactions in the complex. Due to these uncertainties, a structure-based prediction of the binding affinity cannot exceed a certain limit of accuracy [24].

Recently, Kastritis et al reported a dataset containing 144 protein-protein complexes with their corresponding binding affinities [25]. This dataset is larger than the previously available datasets and contains only binding affinity data measured with precise experimental techniques such as SPR, ITC, and fluorescence, thus improving the accuracy of the provided K_d_ values. An additional advantage of this dataset is that the structural information is provided not only for the bound complex but also for the free components, allowing calculation of conformational changes associated with binding. Three recent studies used this benchmark to develop new computational methods for prediction of binding affinity and reported good correlation with experimental data, especially when utilizing a very large number of molecular descriptors [24], [26], [27]. However, machine-learning-based predictors are complex and thus do not provide better understanding of fundamental forces that determine protein binding affinity. In addition, such predictors are biased towards a particular dataset and are likely to perform worse on different datasets.

The goal of the present study is to examine how several types of structure-derived molecular features influence binding energetics and to define particular features that can distinguish between high-, medium- and low-affinity PPIs with statistical significance. Using the Kastritis dataset [25] we construct our own database where for each PPI we calculate a number of biophysical features from the atomic coordinates of the protein-protein complexes and the unbound structures. We explore how well each calculated biophysical feature alone correlates with binding affinity and determine the features that could be used to distinguish between high-, medium- and low- affinity PPIs. Furthermore, we test how various combinations of features could be applied to predict binding affinity and observe some improvement in correlation if more features are incorporated into the equation. In addition, we see a considerable improvement in predictions if we exclude from our analysis low-resolution and NMR structures, revealing the importance of capturing exact intermolecular interactions in our analysis.

## Methods

All the data used for this work is organized in a database which includes all parameters at the atomic level. Different physical features were calculated from PDB files and included in the database.

### Database preparation

144 structures of protein-protein complexes and the corresponding structures of the unbound proteins were extracted from PDB according to the list published in the benchmark database [25]. Complexes with heteroatoms closer than 1.4 Å from the interface were discarded (PDB IDs 1BJ1, 1F34, 1JIW, 1JMO, 1S1Q, 1XD3 and 2J0T). In addition, we excluded a complex where an N-terminal tail close to the binding interface is missing from the structure but is strongly influencing the affinity measurements (PDB ID:2TGP), a complex where two different interfaces are too close to each other to analyze them independently (PDB ID:1NVU), and a complex that has been reported to exhibit an exceptionally high level of disorder-to-order transition [26] (PDB ID:2OZA).

Hydrogens were added to all files with the Reduce software [28] with histidines, asparagines and glutamines allowed to flip. The binding interface for each PPI was defined as all the atoms on one chain that are within 4.8 Å from the second chain in the complex.

### Calculation of difference in accessible surface area (ΔASA)

We implemented the Lee-Richards molecular surface definition [29] using a probe radius of 1.4 Å for the calculation of the accessible surface area at the atomic level. Interface ASA is defined as the area of the atoms that make up the surface of the binding interface. ΔASA is defined as ASA that becomes inaccessible to solvent upon binding.

For each atom in the interface, we defined a *periphery index* as a distance from the closest atom on the surface of the protein that is not part of the interface. Atoms with a *periphery* index ≤3 Å were defined as peripheral atoms. Polar and non-polar area was calculated by summing up the areas of polar and non-polar interfacial atoms.

### Hydrogen bonds

An angle- and hybridization-dependent 12–10 H bond potential [30] with hydrogen bond equilibrium distance of 2.8 Å and a well-depth of 8 kcal/mol was used to calculate the hydrogen bond energy for each pair of potential donor and acceptor atoms [31]. If the calculated H bond energy was lower than −0.6 kcal/mol a satisfied hydrogen bond was counted, otherwise an unsatisfied bond was counted. In the hydrogen bond analysis, we report the number of non-peripherial H bonds, involving the atoms with a periphery index of >3 Å. When an interfacial atom participates in a H bond with another atom on the same protein, an intramolecular H bond was counted. Intermolecular H bonds were counted when a H bond involves a donor and an acceptor atoms that belong to different chains.

### Geometric complementarity

Van der Waals (VdW) interactions were calculated according to the Lennard-Jones 12-6 potential with softened repulsive term [31], [32]. Only neighbor atoms of less than 9 Å were considered for this calculation. VdW interactions were measured between atoms of different chains in the complex. Well depth was set to 0.001 Å when atoms formed a H bond. Other methods used for geometric complementarity calculation in this work include Sc [33], implemented in the Rosetta software [34], and the Katchalsky-Katzir method [35].

### Cavities

Empty spaces within the interface with sufficient volume to accommodate a water molecule were defined as cavities. We then selected only closed cavities that were at a distance of at least 2.8 Å from the surface (corresponding to the diameter of a water molecule). Cavities were detected and their volume was calculated using a simple Monte Carlo Integration technique [36].

### Conformational changes

Structures of the unbound proteins were superimposed onto the structure of the complex [37]. Root Mean Square Deviation (iRMSD) between bound and unbound structures was calculated for all Cα atoms belonging to the interface.

To calculate side chain conformational changes upon binding, we assigned χ_1_ and χ_2_ dihedral angles to all side chains in the binding interface for the bound and the unbound structures. When at least one of these angles differed by more than 20 degrees between the unbound and the bound protein, the residue was considered to change conformation upon binding. The number of residues that do not change conformation upon binding was normalized by the total number of residues in the interface to give a percentage of residues that do not change side chain conformation. For NMR structures, only the first model was analyzed.

### Hot spots

*In silico* alanine scanning was performed with the Robetta server that uses a fixed backbone approximation and an energy function parameterized on Ala mutations [38]. We defined hot-spot positions where mutation to Ala destabilized the complex by at least 1 kcal/mol.

### Binding Interface composition

The *interface propensities* for each of the 20 natural amino-acids, for each PPI, were calculated as a ratio between ASA that the amino acid contributes to the interface and ASA that it contributes to the whole surface according to.the equation: AA_propensity_  =  (ASA AA_i_/ASA interface)/(ASA AA_s_/ASA surface). Here ASA AA_i_ is the surface area of a particular amino acid in the interface and ASA AA_s_ is the surface area of the same amino acid on the whole surface of the protein [5]. The results were averaged over the entire database for each amino acid. *High- and Low-affinity propensities* for each amino acid were calculated similarly as above but using only PDBs belonging to high- and low-affinity groups, respectively.

### Electrostatic interactions

Salt bridges were counted according to Xu et al [11]. When distance between charged atoms is less than 4 Å and they participate in a hydrogen bond, a salt bridge is counted. Electrostatic energy was calculated using a Coulomb equation with a dielectric constant of 10 [31] and using the Delphi software [39] that utilizes linear Poisson-Boltzmann equation. For the latter calculation we used interior and exterior dielectric constants of 2 and 80, respectively and salt concentration of either 0 or 0.145 M. The electrostatic component of the binding free energy was obtained as the difference between the electrostatic free energy of the complex and those of the unbound chains.

### Receiver Operator Characteristic (ROC) plots

For every tested biophysical feature we drew a ROC plot. This plot goes through all possible cut-off values that are used to distinguish between two groups, e. g. high- and low-affinity PPIs. Y-axis shows the fraction of PPIs from high-affinity PPIs that are above the cutoff values, as expected (true positive rate). X-axis shows the fraction of PPIs that are above the cut-off value in spite of belonging to low-affinity PPIs (false positive rate). Both x and y are normalized to 1 and the area under the curve (AUC) represents the performance of prediction when distinguishing between the two groups using the particular feature.

### Combining biophysical features for affinity prediction

We considered all possible combinations of thirteen different biophysical features that are likely to be independent of each other (see table S2). Using a linear combination of all considered features, we optimized the weights in front of each feature to get the best correlation with experimental binding affinity. This optimization was performed starting from all combinations of two features and finishing with all possible combinations of thirteen features. Using the optimized weights, we calculated Pearson's correlation coefficient R and the AUC for ROC plots for high- vs. low-, high- vs. medium-, and medium- vs. low-affinity PPIs. Finally, the best values of R, and AUCs for each number of considered features were plotted. Models were cross-validated using the leave one out algorithm when the number of samples was below 100. This method requires fitting the data N times for a dataset of N points. In each round of fitting, one file is excluded from calculations and its affinity is predicted with the best weights in front of each feature. The N predicted values are then compared to the experimental values and the Pearson correlation coefficient R is reported.

## Results and Discussion

To achieve the best possible accuracy in our analysis, we first systematically inspected all PPIs presented in the Kastritis database. We decided to exclude complexes that contain heteroatoms in the vicinity of the binding interface since the effect of heteroatoms on binding affinities cannot be accurately predicted. In addition, we excluded a complex where an N-terminal tail close to the binding interface is missing from the structure but is strongly influencing the affinity measurements (PDB ID:2TGP), a complex where two different interfaces are too close to each other to analyze them independently (PDB ID:1NVU), and a complex that has been reported to exhibit an exceptionally high level of disorder-to-order transition [26] (PDB ID:2OZA). After these exclusions we were left with 133 PPIs in our dataset. Each PPI was assigned into one of the three groups of high (Kd ≤10^−9^ M), medium (10^−9^ M <Kd ≤10^−6^ M) and low affinities (Kd>10^−6^ M) containing 43, 60, and 30 complexes in each group (see Figure S1 for distribution of datapoints). In addition, we divided the complexes into rigid and flexible according to the extent of conformational changes they exhibit upon binding. Complexes were defined flexible if the Root Mean Square Deviation between interfacial Cα atoms (iRMSD) of the bound and the unbound structures was ≥1 Å and were defined as rigid otherwise. For each PPI in our dataset, the binding interface was defined and was broken down into atoms with assigned coordinates and chemical properties. For each complex, we calculated a number of biophysical features that we considered important for determining binding affinity. These features included accessible surface area, inter- and intra-molecular H bonds, changes in main chain and side chain conformations between the bound and unbound structures, geometric complementarity, electrostatic interactions, the number of hot-spots, interface composition and volume of cavities. Some features were calculated with a number of different methods and the results were compared. Finally, using the Receiver Operator Characteristic (ROC) analysis, we compared how different features could be used to distinguish between low-, medium-, and high-affinity complexes.

### Changes in the accessible surface area (ΔASA)

**Figure 1:**
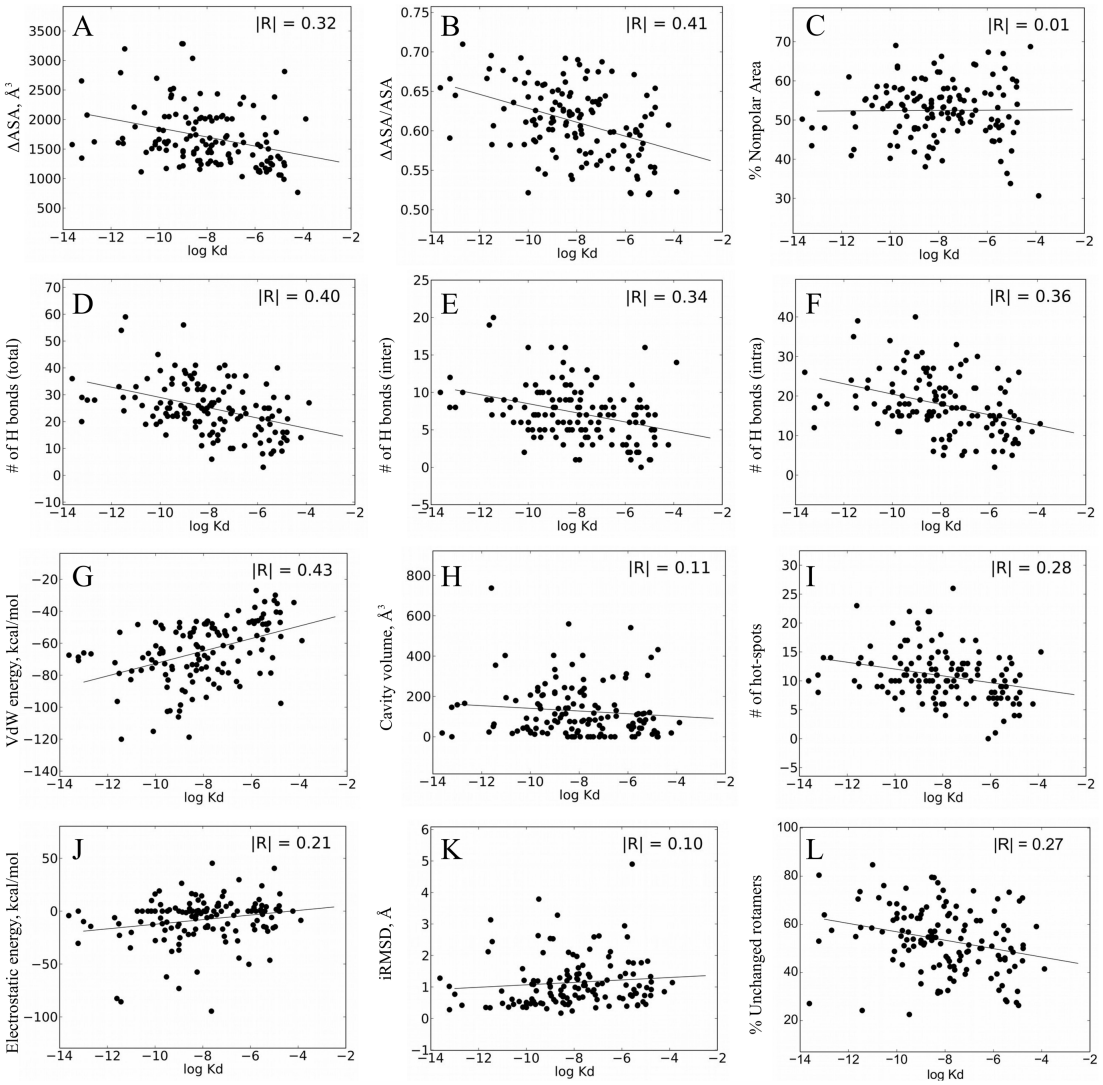
Dependence of Kd on various single biophysical features. (A) Change in the accessible interface surface area (ASA); (B) ΔASA normalized to the total interface area; (C) percent of non-polar change in the accessible surface area; (D) the total number of interfacial H bonds, (E) the number of intermolecular interfacial H bonds, (F) the number of intra-molecular H bonds; (G) Van der Waals energy; (H) volume of cavities; (I) number of hotspots; (J) electrostatic columbic energy; (K) RMSD between bound and unbound structures for interface Cαs; (L) percentage of rotamers that do not change conformation upon binding. Each point represents one PDB file in the database and the line corresponds to a linear fit to all data points in the database.

ΔASA is the total area that gets buried upon the complex formation. Earlier studies reported that protein binding affinity depends on ΔASA, with high-affinity complexes burring more surface area [5], [19], [40]. We found that ΔASA exhibits some correlation with binding affinity, with an R-value of 0.32 (Figure 1A). A better correlation (R = 0.41) is obtained if we normalize ΔASA by the total area of the atoms in the binding interface, providing a measure of binding interface dehydration (Figure 1B). The moderate correlation between ΔASA and affinity is not surprising since it is known that a few point mutations can produce many-fold changes in binding affinity without significant changes in ΔASA. For example, cognate and non-cognate complexes of colicin/immune proteins exhibit affinities of 10^−14^ and 10^−8^ M respectively while showing very similar ΔASA [41]. Furthermore, a single mutation in hemagglutinin from Influenza virus reduces the affinity of this protein for an antibody from 10^−9^ to 10^−6^ M [42]. Affinity maturation experiments in many antibody-antigen complexes also argue against strong correlation between ΔASA and binding affinity [43]. Thus, PPIs with larger ΔASA do not necessarily exhibit higher affinity but have a potential for containing more productive intermolecular interactions and for achieving higher affinity through mutations.

In some protein design studies, binding affinities of PPIs were enhanced by substituting polar residues with hydrophobic amino acids, implying that non-polar buried surface area should correlate with affinity [44]. We thus decided to examine whether polarity of the binding interface is correlated with binding affinity in our dataset. We found no correlation between binding interface polarity and affinity (Figure 1C) with PPIs exhibiting on average 48:52 ratio between polar and hydrophobic surface area, with a standard deviation of 7%. We conclude that binding interfaces of high-affinity complexes are not more hydrophobic than those of low-affinity complexes. This lack of correlation between polarity and affinity might be a result of the evolutionary pressure to keep protein surfaces mostly polar, preventing protein aggregation.

### H bonds

H bond interactions are crucial to both protein folding and binding. While the energy of one H bond is relatively small, a large number of H bonds in proteins make them significant contributors to protein energetics. We hence calculated the number of H bonds formed across the binding interface (intermolecular H bonds), and within the interface (intramolecular H bonds) for the complexes in our dataset. Here we excluded H bonds that lie on the periphery of the binding interface since buried H bonds were expected to be more important for binding energetics compared to exposed H bonds. We also calculated the number of unsatisfied H bonds (the number of H bond donors that are buried within the interface and do not participate in H bonds). Unlike some previous studies [5] our analysis was based on calculating the energies of the H bonds according to atomic distances and angles between the donor, the hydrogen, and the acceptor atoms (see Materials and Methods). Our results show that the highest correlation (R = 0.40) is observed between affinity and the total number of H bonds in the binding interface (Figure 1D). Slightly lower correlation is observed when considering inter- or intra-molecular H bonds alone with R-values of 0.34 and 0.36, respectively (Figure 1E and 1F). Our results are in agreement with the general notion that intermolecular H bonds improve the enthalpic term of the free energy of binding, while the intramolecular bonds stabilize the interface and thus reduce the unfavorable entropic change associated with binding. Unexpectedly, no correlation with binding affinity is observed for interfacial unsatisfied H bonds for all PPIs in the dataset (Figures S2).

### Geometric complementarity

Protein-protein recognition has been first described by the lock-and-key theory developed already hundred years ago [45]. This model assumes complete geometric complementarity of the two binding interfaces and does not consider any conformational change upon binding. Geometric complementarity in binding interfaces is hence a likely parameter for determining PPI binding affinity. We calculated geometric complementarity using three different methods including Katzir's molecular surface recognition [35], surface complementarity parameter Sc [33] and Van der Waals energy [30]. The best correlation with binding affinity is obtained when using VdW energy as a measure of geometric complementarity (Figure 1G). This feature shows an R-value of 0.43. Substantially lower correlation with affinity is observed when using two alternative methods for geometric complementarity calculation (Figures S3A and S3B). When separating between rigid and flexible complexes, Van der Waals energy gives a comparable correlation with binding affinity for both groups while the Katzir method gives much lower correlation for flexible complexes compared to rigid complexes and Sc parameter gives no correlation for either rigid or flexible complexes (Table S1). These results suggest that Van der Waals energy best approximates the energetic contribution of geometric complementarity to protein binding.

### Cavities

Cavities could be observed as packing defects within protein cores or binding interfaces [46], [47]. Cavities in protein cores have been shown to be extremely unfavorable and their removal usually produces high stabilization of the protein [48], [49]. The role of cavities in protein-protein interfaces has been less studied compared to their role in protein cores. Chakrabarti and colleagues report that cavities in interfaces are on average larger compared to cavities in protein cores and their size correlates well with the size of the monomeric protein but not with the size of the interface [50].

We computed the volume of closed cavities in binding interfaces of PPIs in our dataset and examined how this feature correlates with binding affinity. In contrast to our expectations, we observed no correlation between the total cavity volume and binding affinity (Figure 1H). Interfaces of high- and low-affinity complexes exhibit an average total cavity volume of 146 and 104 A^3^ respectively. The lack of correlation may be due to the fact that only empty cavities, not containing water molecules, are destabilizing for binding. However, we could not distinguish between empty and water-containing cavities in our database due to insufficient resolution of some of the crystal structures in our study.

### Hot Spots

It has been observed that a relatively small number of interface residues, referred to as hot-spots, account for the majority of the binding energy [51]. Hot-spot residues are usually defined as binding interface positions where mutations to Ala produce more than 1 kcal/mol destabilization of the complex. We calculated the number of hot-spots for each PPI in the dataset using the Robetta server [52] and explored whether the calculated number of hot-spots in the interface correlates with binding affinity. We found a moderate correlation between affinity and the number of interfacial hot-spots with an R value of 0.28 (Figure 1I). One possible reason for relatively modest correlation between affinity and the number of hot-spots are errors associated with hot-spot predictions due to inaccuracies in the energy function and/or the fixed backbone approximation.

### Electrostatic interactions

Past studies have demonstrated that electrostatic interactions, in general, are destabilizing in protein folding [39], [53] but stabilizing in protein binding [54]. Such difference was explained by more favorable energetic change associated with transferring hydrophilic pairs from the surface of unbound proteins to the complex compared to the same change associated with protein folding. Here, we calculated electrostatic interactions in binding interfaces using two alternative methods, first by simply counting the number of intermolecular salt bridges [11], [55] and second by calculating the exact electrostatic interaction energy [30], [39], [56], [57].

We found that the number of salt bridges as defined by Xu et al [11] does not correlate with binding affinity (Figure S4A). Similar results are obtained if we count repulsive same-charge intermolecular interactions (Figure S4B). Nevertheless, the number of salt bridges shows some correlation with the interfacial area (R-value of 0.44). That is, the number of salt bridges is dependent on the area of the interface, but its contribution to binding energetics is low. We thought that the lack of correlation between electrostatics and the binding affinity might be due to our simplistic approach for approximating this term. We hence performed more rigorous calculations to obtain electrostatic energy between the two protein chains involved in binding using either the Coulombic potential or a more sophisticated Poisson-Boltzmann equation [39], [58]. In both cases, we found no substantial correlation between electrostatic energy and binding affinity (Figure 1J and Figure S4C). These results indicate that both high- and low-affinity complexes could contain electrostatically optimized or non-optimized interfaces.

### Binding interface composition

**Figure 2:**
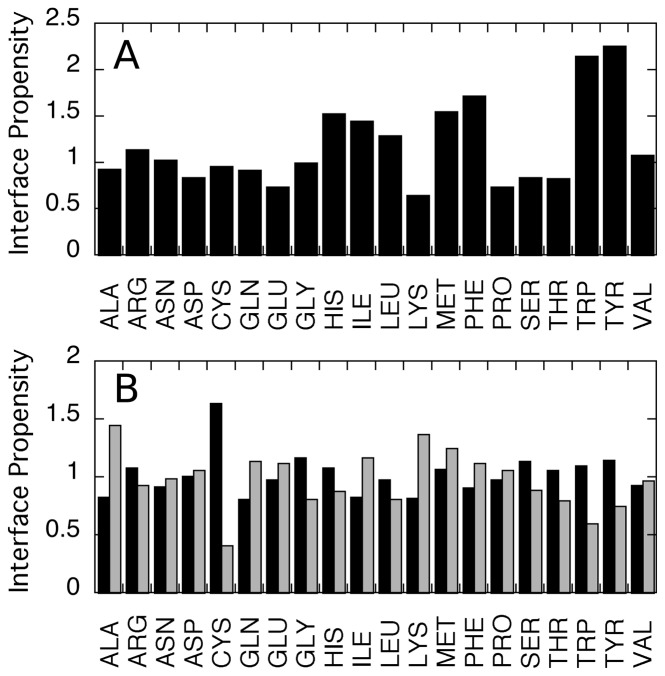
Amino acid interface propensities. (A) Amino acid propensities to be in an interface compared to protein surface calculated according to [5] (B) Amino acid propensities for high-affinity (black) and low-affinity (grey) complexes.

Several recent studies concluded that certain amino acids such as Tyr, Trp, Phe, Met, Val, Cys and Ile appear more frequently in PPI binding interfaces [5]. Other studies however, showed no appreciable difference in the composition of interfaces of the whole genomes [59], [60]. To our knowledge, no study attempted to correlate amino acid composition to binding affinity. We hence examined whether this feature plays a role in determining binding affinity by calculating amino acid propensity to be in an interface [5], first for all complexes in our database and then separately for low- and high-affinity complexes (Figures 2A and B). For all complexes in the database, we observe that Trp, Tyr, Phe and Met are the most frequent interfacial amino acids, in agreement with previous studies. We also observe that Tyr, Trp, His, and Cys have a higher propensity to be found in high-affinity interfaces compared to low-affinity interfaces. However, due to a relatively small number of data points, these results are not statistically significant, pointing to the necessity of enlarging the dataset under study. Interestingly, Ala propensity shows an anti-correlation with affinity that is highly significant, indicating that this amino acid, within an interface, cannot provide favorable interactions. Lys also shows higher propensity to be found in low-affinity complexes compared to high-affinity complexes, in agreement with a previous study [61].

High propensity of Cys in high-affinity interactions could be partially explained by the fact that many of the high-affinity PPIs are enzyme-inhibitor complexes and inhibitors frequently contain multiple cysteins, most of them linked in intramolecular disulphide bonds. Indeed, in high-affinity complexes, Cys residues are mostly oxidized exhibiting an 8:1 oxidized/reduced ratio, while in low-affinity complexes this ratio reduces to 1:1. We thus conclude that cysteins bring rigidity to the main chain of the interface, lowering the entropic cost of binding.

### Conformational changes upon binding

Protein-protein binding is a thermodynamic process that involves gain of favorable enthalpy and unfavorable reduction in entropy upon complex formation. However, the entropic term of binding is difficult to calculate and has been estimated only in a few studies [5], [25], [62], [63], [64]. The Kastritis database [25] allows for estimation of the entropic term by analyzing conformational changes associated with binding on both the backbone and the side chain level.

We first analyzed backbone conformational changes associated with binding by calculating iRMSD between Cα atoms of the bound and the unbound conformations. For all complexes in the database, no measurable correlation was observed between binding affinity and iRMSD (Figure 1K). However, when analyzing only rigid complexes (N = 69) we observed that iRMSD decreases with increased affinity with a correlation of R = 0.35 (Figure S5). This result indicates that iRMSD is a good measure of contribution of conformational movements to binding energetics when such movements are small. However, when large conformational changes are involved iRMSD becomes a poor measure of binding affinity.

We next measured side chain conformational rearrangements for interface residues due to binding by calculating the differences in χ1 and χ2 angles between the bound and the unbound states. We further correlated percentage of side chains that did not change conformation upon association to binding affinity. We found that when all complexes are considered, the correlation between percentage of residues that do not change conformation and affinity is moderate (R = 0.27, Figure 1L). This value however increases substantially to 0.51 if we restrict our analysis to high-resolution X-ray structures (see section on high-resolution structures). Previous reports demonstrated that some key side chains in binding interfaces are pre-oriented for binding [20], [65]. Our results corroborate these findings and further reveal that high-affinity complexes are more likely to have their side chains pre-orientated for binding compared to low-affinity complexes. Such side chain pre-orientation minimizes the entropy loss upon binding and thus increases affinity. This feature however, could not be computed accurately for low-resolution structures.

### High-resolution structures show better correlation to binding affinity for most of the features

**Figure 3:**
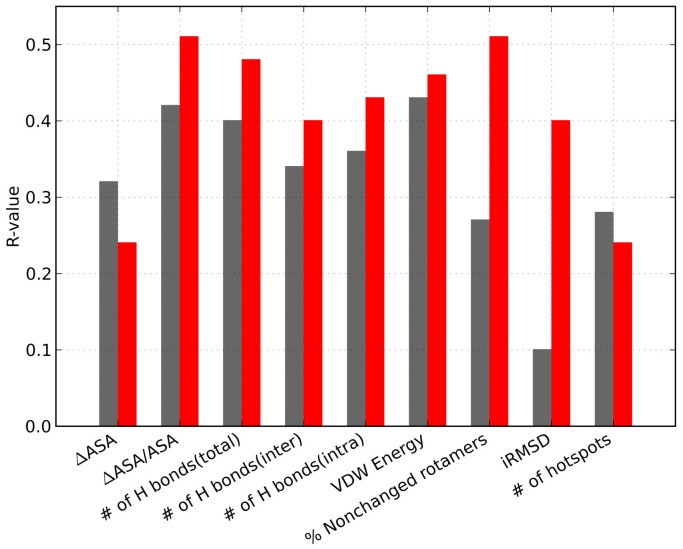
Improvement in R-value for high-resolution structures. Barplot displaying correlation (R-value) between different biophysical features and K_d_ when using only high-resolution structures (red bars) and all structures (grey bars).

We thought that modest correlation with binding affinity for most biophysical features could be in part due to the limited resolution of the structures under study. We hence compared the R-values calculated for all structures in the dataset to those calculated for only high-resolution files (X-ray structures with a resolution <2.5 Å for both the complex and the free components, 37 PPIs). To exclude possible bias coming from lowering the number of samples in our analysis, we performed the same calculation using only low-resolution structures in our dataset. Interestingly, we find that most features show a considerable improvement in R-values when only high-resolution structures are considered (Figure 3). In contrast, decrease or no change in R-value was observed for most features for low-resolution structures (Figure S6). Very substantial improvement in R-values for high-resolution structures is shown for iRMSD and the percentage of side chains that did not change conformation upon binding (corresponding to 0.40 and 0.51, respectively), indicating that these features are most sensitive to resolution of the structure. Overall, our analysis shows that low resolution of structures is a limitation that results in inaccuracies of binding affinity predictions and some biophysical descriptors are more sensitive than others to such inaccuracies.

### Distinguishing between low-, medium-, and high-affinity complexes

**Figure 4:**
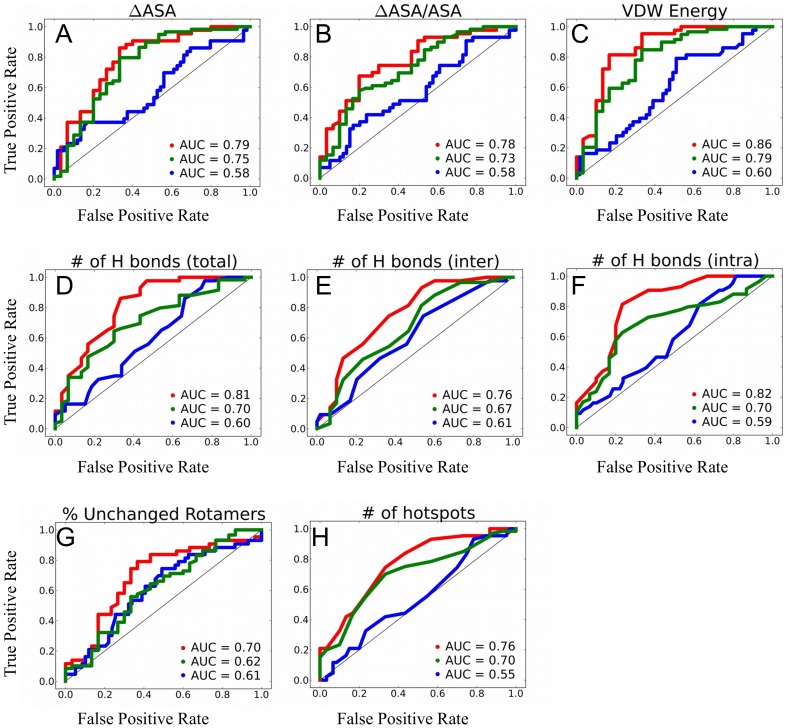
Receiver Operator Characteristic Analysis. The graph shows the true positive rate vs. false positive rate in discriminating high- from low-affinity PPIs (red line), medium- from low-affinity PPIs (green line) and high- from medium-affinity PPIs (blue line) for each feature. Each point represents a particular cut-off value used to discriminate between the two groups. Features included in the figure are (A) ΔASA, (B) ΔASA/ASA, (C) Van der Waals energy, (D) the total number of interfacial H bonds, (E) the number of intermolecular interfacial H bonds, (F) the number of intra-molecular H bonds; (G) Percentage of rotamers that do not change conformation upon binding; and (H) the number of hotspots.

Using ROC analysis, we tested how each of the explored biophysical features can distinguish between high-, medium-, and low-affinity PPIs. In this analysis we included only thirteen features that showed some correlation with binding affinity (Table S2). Our results show that several single features could distinguish well between high- and low-affinity PPIs with Area Under the Curve (AUC) values reaching 0.86 and 0.81, for the VdW energy and the total number of H bonds, respectively (Figure 4, red curves). Slightly lower AUC values are obtained when distinguishing between medium- and low-affinity complexes (Figure 4, green curves). However, worse results are obtained when distinguishing high-affinity complexes from medium-affinity complexes, pointing to apparent similarity of these two groups (Figure 4, blue curves). Inability to discriminate between these two groups of PPIs is probably due to the composition of out database that contains many PPIs with a K_d_ near the cutoff between high- and medium-affinity groups (Figure S1).

**Figure 5:**
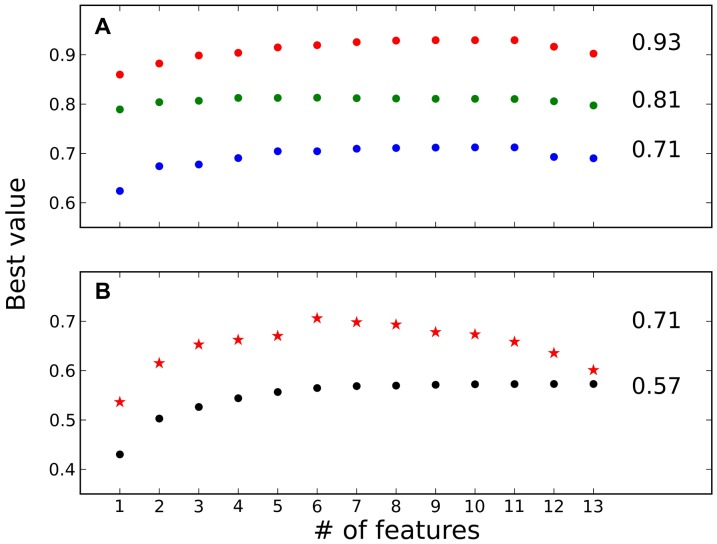
Incorporating more features in the prediction improves correlation with K_d_ and ROC analysis. The best possible weights were obtained to combine the features into one equation using a linear fit to the experimental data. X-axis shows the number of features used to predict K_d_ and to discriminate between the two groups. Y-axes shows the best value obtained for each number of features used in the equation. The analysis was performed on all structures in the database (filled circles) and on high-resolution structures only (red stars). (A) AUC were evaluated on high- vs low-affinity (red), medium- vs low-affinity (green) and medium- vs high-affinity (blue) PPIs (B) Pearson's correlation coefficient for all dataset (filled circles) and for high-resolution structures only (red stars).

We further explored whether our predictions could be improved by combining several features into one formula (see Methods). Figure 5A and B shows that both AUC and R-values could be increased if several features are combined in one linear equation reaching the maximum R-value of 0.57 and maximum AUCs of 0.93, 0.81 and 0.71 for discriminating between high- and low-, medium- and low- and high- and medium-affinity PPIs, respectively. However, introduction of more than four features does not improve predictions substantially, indicating that the considered features are probably interdependent and/or overfitting is observed. Finally, when we restrict our dataset to high-resolution structures, the correlation with binding affinity significantly improves for all predictions using combinations of features reaching a maximum R-value of 0.71 (Figure 5B, red stars).

## Conclusions

In this work we tested how a number of features derived from a PPI structure correlate with PPI binding affinity. The features that showed the highest correlation with binding affinity are the total number of H bonds, geometric complementarity measured by the van der Waals energy, and side chain conformational changes for high-resolution X-ray structures. In spite of moderate correlation and high variability, a number of tested single biophysical features could be used to discriminate between high- and low-affinity complexes as well as between medium- and low-affinity complexes with high significance and hence could be used to predict the range of affinities from structure. These features include not only those determining enthalpic contribution to binding but also those directly related to the entropic term such as iRMSD, change in side chain conformation, the number of disulfide bonds and intramolecular H bonds. Correlation with binding affinity for most of the studied features could be improved by restricting the analysis to high-resolution X-ray structures. Combining several biophysical features into one equation results in further improvement of our predictions and allows for unequivocal discrimination between high-, medium- and low-affinity PPIs. Finally, incorporating the information for both bound and unbound states improves the accuracy of the binding affinity predictions and could be utilized for developing new energy functions for design of PPIs.

## Supporting Information

Figure S1 **Density estimation of logKd.** Kernel density estimation of the probability density function of the logKd for the whole dataset. Each circle represents one pdb file in the dataset.
(TIFF)

Figure S2 **Unsatisfied Interfacial H bonds.** Number of unsatisfied hydrogen bonds vs. Kd. Each point represents one PDB file in the database.
(TIFF)

Figure S3 **Geometric complementarity vs. Kd.** Surface complementarity calculated using the Katzir score (A) and the Sc core (B) vs. Kd. Each point represents one PDB file in the database and the line corresponds to a linear fit to all data points in the database.
(TIFF)

Figure S4 **Electrostatic interactions vs. Kd.** (A) the number of interfacial salt bridges, (B) the number of repulsive electrostatic interactions and (C) the intermolecular Coulombic electrostatic energy calculated with the Poisson-Boltzmann equation vs. Kd. Each point represents one PDB file in the database and the line corresponds to a linear fit to all data points in the database.
(TIFF)

Figure S5 **iRMSD vs. Kd.** iRMSD of rigid structures (iRMSD ≤1) vs. Kd. Each point represents one PDB file in the database and the line corresponds to a linear fit to all data points in the database.
(TIFF)

Figure S6 **R-values for All and low-resolution structures.** Barplot displaying the correlation of different PPI features to affinity using only low-resolution structures (blue bars) compared to all structures in the database (grey bars). On the y-axis, the Pearson's correlation to affinity. On the x-axis, the different features whose correlation to affinity was measured.
(TIFF)

Table S1 **Pearson correlation coefficient R and p-values for the three methods used for surface complementarity analysis computed over the entire database, rigid, and flexible complexes.**
(DOC)

Table S2 **List of the 13 different biophysical features that were considered in a linear combination to fit experimental Kd values. linear combination to fit experimental Kd values.**
(DOC)
