# Supplementary material for: How Structure Defines Affinity in Protein-Protein Interactions
Source: PLoS One. 2014 Oct 16;9(10):e110085. doi: 10.1371/journal.pone.0110085 (PMC4199723; doi:10.1371/journal.pone.0110085)
Supplement: Table S1 — Pearson correlation coefficient R and p-values for the three methods used for surface complementarity analysis computed over the entire database, rigid, and flexible complexes. (DOC) [file pone.0110085.s007.doc]

**Table S1**. Pearson correlation coefficient R and p-values for the three methods used for surface

complementarity analysis computed over the entire database, rigid, and flexible complexes.

|  | VdW^a^ | | | Katzir^b^ | | | | Sc^c^ | | | |
| --- | --- | --- | --- | --- | --- | --- | --- | --- | --- | --- | --- |
|  | All | Flexible^d^ | Rigid^e^ | | All | Flexible^d^ | Rigid^e^ | | All | Flexible^d^ | Rigid^e^ |
| R | 0.43 | 0.38 | 0.55 | | 0.23 | 0.14 | 0.36 | | -0.01 | 0.15 | -0.13 |
| p-value^f^ | 1x10^-7^ | 3x10^-4^ | 4x10^-6^ | | 1.9x10^-3^ | 0.1 | 2.0x10^-3^ | | 0.4 | 9.8x10^-2^ | 0.1 |

^a^Energy of the Van der Waals interactions [31,32]

^b^Katzir's method [35]

^c^Sc method [33]

^d^complexes with iRMSD ≥ 1 A

^e^complexes with iRMSD < 1 A.

^f^p-values are calculated comparing high- and low-affinity complexes in all the database, and for

flexible and rigid subgroups
