# Supplementary material for: How Structure Defines Affinity in Protein-Protein Interactions
Source: PLoS One. 2014 Oct 16;9(10):e110085. doi: 10.1371/journal.pone.0110085 (PMC4199723; doi:10.1371/journal.pone.0110085)
Supplement: Table S2 — List of the 13 different biophysical features that were considered in a linear combination to fit experimental Kd values. linear combination to fit experimental Kd values. (DOC) [file pone.0110085.s008.doc]

**Table S2.** List of the 13 different biophysical features that were considered in a linear combination to fit experimental Kd values.

1. ∆ASA

2. ∆ASA/ASA

3. ∆ASA of the polar area

4. ∆ASA of the non-polar area

5. ∆ASA of the aromatic area

6. Number of intra H bonds

7. Number of inter H bonds

8. VdW energy

9. % Unchanged Rotamers

10. iRMSD

11. Number of hot-spots

12. volume of cavities

13. Electrostatic Coulomb energy
